# Akt2 knock-down reveals its contribution to human lung cancer cell proliferation, growth, motility, invasion and endothelial cell tube formation

**DOI:** 10.1038/srep12759

**Published:** 2015-08-03

**Authors:** Samir Attoub, Kholoud Arafat, Nasseredine Kamel Hammadi, Jan Mester, Anne-Marie Gaben

**Affiliations:** 1 Department of Pharmacology & Therapeutics, College of Medicine & Health Sciences, UAE University, P. O. Box: 17666, Al Ain, United Arab Emirates; 2 INSERM U673 and U938, Molecular and Clinical Oncology of Solid Tumors, University Pierre and Marie Curie Paris VI, Saint-Antoine Hospital, 75571 Paris Cedex 12, France

## Abstract

The Akt/PKB serine/threonine protein kinase consists of three isoforms: Akt-1, −2 and −3. Their overexpression has been detected in human cancers, but their roles in cancer progression are unclear. We investigated the impact of specific silencing of Akt1 and Akt2 on human lung cancer cell proliferation, colony growth, motility, and invasion *in vitro* as well as tumor growth *in vivo* using human Non-Small Cell Lung Cancer cells LNM35, and on the vascular tube formation using HUVEC cells. Although silencing of Akt1 decreased cellular invasion at least in part via COX-2 inhibition, it had almost no effect on cell motility, proliferation, colony formation, and angiogenesis. Transient as well as stable silencing of Akt2 resulted in a strong inhibition of Rb phosphorylation associated with a decrease in cellular proliferation and colony formation, leading to the inhibition of tumor growth in the xenograft model. Silencing of Akt2 also reduced cellular motility and invasion *in vitro*, presumably via COX-2 inhibition. Moreover, silencing of Akt2 in the HUVEC cells resulted in the inhibition of their spontaneous angiogenic phenotype. Altogether, these results indicate that Akt2 plays an important role in lung cancer progression and can be a promising target for lung cancer therapy.

Akt, also known as protein kinase B (PKB), is a family of three serine/threonine kinases Akt1/PKBα, Akt2/PKBβ, and Akt3/PKBγ. Activation of Akt appears to play crucial roles in metabolism, cellular growth and survival. Akt is activated by several growth factors, including epidermal growth factor (EGF), insulin, insulin-like growth factors (IGFs), and leptin^1^,^2^, *via* the 3-phosphoinositide-dependent protein kinase-1 (PDK1)/PI3K pathway. In addition, the rictor-mTOR complex directly phosphorylates Akt/PKB on Ser473 and facilitates Thr308 phosphorylation and Akt activation by PDK1^3^. In genotoxic-stressed cells, DNA-PK is also responsible for the phosphorylation of PKB/Akt on Ser473 in the DNA repair signaling pathway^4^. Activated Akt in turn phosphorylates and inhibits GSK3β, leading to increased stability of cyclin D1 and c-Myc, two critical mediators required for cell cycle progression^5^. Parallel to the Ras/MAPK pathway, the PI3K-Akt signaling cascades regulate cell cycle progression at the G1/S transition. In addition, Akt protects cells against apoptosis *via* phosphorylation of the IκΒ kinase leading to the activation of the NFκΒ survival factor, and inactivation of several pro-apoptotic factors, including BAD and caspase-9^6^,^7^. As a consequence, Akt promotes tumor resistance to cancer chemotherapy and radiotherapy^8^,^9^. Besides, accumulating evidence implicates the PI3K-Akt pathway in the regulation of cancer cell motility, tumor invasion and metastasis^10^,^11^.

All these functions of Akt make this signalling element an attractive target for cancer therapy^11^,^12^. It has been established that the Akt cascade is linked to the actions of c-src, c-kit, c-met and other transforming pathways initiated by the HER and IGF receptors. Accordingly, the anticancer activity of several humanized function-blocking antibodies and tyrosine kinase inhibitors such as Herceptin and Gleevec, respectively targeting ErbB2/HER2 and abl/c-kit, rely at least in part on their impact on the PI3K-Akt pathways. In line with this proposition, Akt overexpression and constitutive activation have been demonstrated in premalignant and malignant human bronchial epithelial cells^9^,^13^,^14^. Similar observations were made in several established solid tumors of the urogenital and digestive systems^15^,^16^,^17^.

The three Akt isoforms Akt1, −2, −3 are ubiquitously expressed in normal and tumor tissues^17^,^18^. Compared to Akt1, Akt2 is abundant in insulin-responsive tissues^19^. Akt3 isoform is predominantly expressed in brain, heart, kidney, lung, breast, prostate, and colon^17^,^20^. Akt2 and Akt3 share respectively 81 and 83% primary sequence homology with Akt1, suggesting overlapping signaling functions for the three Akt isoforms. However, the degree of functional redundancy between Akt1, Akt2, and Akt3 in cancer cell survival, proliferation and invasion remains unclear. Identification of a given Akt isoform as the most preferred target in human cancer therapy is still an unanswered question, and would be important in order to avoid unnecessary unwanted side effects. Using RNA interference selectively targeting Akt1 and -2 isoform, we explored their respective roles in the human lung cancer cells’ proliferation and colony growth *in vitro* and in tumor growth *in vivo* as well as its role in cell motility and invasion. Their role in angiogenesis was explored *in vitro* using human umbilical vein endothelial cells.

## Materials and Methods

### Cell culture, antibodies, siRNA and shRNA

LNM35 (NCI-H460-LNM35) is a highly tumorigenic, invasive and metastatic large cell lung carcinoma^21^. LNM35 and A549 human lung cancer cells were maintained in RPMI 1640 (Invitrogen, Paisley, UK), human mammary adenocarcinoma cells MDA-MB-231 and MCF-7, and human colon cancer cells HT-29 were maintained in DMEM (Invitrogen, Paisley, UK). All media were supplemented with antibiotics (penicillin 50 U/ml; streptomycin 50 μg/ml) (Invitrogen, Cergy Pontoise, France) and with 10% fetal bovine serum (FBS, Biowest, Nouaille, France). EndoGROTM Human Umbilical Vein Endothelial Cells (HUVECs) (Millipore, Temecula, CA) were maintained in EndoGROTM-MV-VEGF Complete Media Kit (Millipore, Temecula, CA).

Anti-Akt1 (2H10) mouse mAb, anti-Akt2 (5B5) rabbit mAb, and Phospho-Rb (Ser807/811) (D20B12) XP® Rabbit mAb were obtained from Cell Signaling Technology (Beverly, MA) and COX-2 mouse monoclonal antibody, Rb (C-15) rabbit polyclonal antibody, β-actin (sc-1615-HRP) polyclonal antibody from Santa Cruz Biotechnology (Santa Cruz, CA, USA). The siRNA transfection reagent used was Dharma*FECT* (Dharmacon, Lafayette, USA). Control siRNA and siRNA targeting Akt1 and Akt2 were synthesized by Eurogentec (Liege, Belgium)^22^. The second set of control and Akt1 and Akt2 siRNA duplexes were synthesized by Dharmacon (Thermo Fisher Scientific, Dharmacon Products, Lafayette, CO, USA). SMARTvector 2.0 Lentiviral shRNA particles (Dharmacon Thermo Scientific, US) bind to cells and deliver their genetically engineered RNA genome to the cytoplasm. The SMARTvector 2.0 includes a turboGFP reporter gene to facilitate assessment and optimization of transduction efficiencies. This vector also contains a puromycin resistance gene for selection and isolation of clonal populations when generating stable cell lines.

### Transient and stable silencing of Akt1 and −2 in LNM35 cells

For transient transfection, cells were seeded in 35 mm Petri dishes (5 × 10^4^ cells) and allowed to attach for 24 h. Transfections were carried out in 1 ml Opti-MEM using Dharma*FECT* siRNA transfection reagent (Thermo Fisher Scientific, Dharmacon Products, Lafayette, CO, USA), according to the manufacturers’ protocols. After 6 h incubation with the Akt1 and Akt2 siRNA or the synthetic control oligonucleotides (100 nM final concentrations), 1 ml of 10% FBS containing medium was added. After 48 h, 72 h and 120 h following transfections, cells were harvested and lysed for Western blot analysis.

For stable Akt1 and Akt2 silencing, cells were seeded at a density of 20,000 cells/well into 96-well plates and allowed to attach for 24 h. Cells were transduced with SMARTvector 2.0 Lentiviral shRNA particles targeting Akt1, Akt2 or SMARTvector 2.0 Non-Targeting control particles (Dharmacon Thermo Scientific, US). Selection of cells stably expressing Akt1-, Akt2-shRNA and the Control-shRNA started 72 h post-transfection. Growth medium was replaced with fresh selection medium containing 10 μg/mL of puromycin. Puromycin-containing medium was refreshed every 2–3 days, and selection of stable cells expressing Akt1-shRNA, Akt2-shRNA or Control-shRNA was completed after approximately 4 weeks. Multiple clones and pools of clones were expanded, harvested, and prepared for western blot analyses.

### Western Blot analysis

Total cellular proteins were isolated using RIPA buffer (25 Mm Tris.HCl pH 7.6, 1% nonidet P-40, 1% sodium deoxycholate, 0.1% SDS, 0.5% protease inhibitors cocktail (Sigma, Steinheim, Germany), 1% PMSF, 1% phosphatase inhibitors cocktail (Thermo Scientific, Rockford, USA). The whole cell lysate was recovered by centrifugation at 14,000 rpm for 20 minutes at 4 °C to remove insoluble material and 25–50 μg of proteins were separated by SDS-polyacrylamide gel electrophoresis. After electrophoresis, the proteins were electro-transferred onto a nitrocellulose membrane, blocked with 5% non-fat milk and probed with Akt1, Akt2, Rb, Phospho-Rb, COX-2, and β-actin antibodies overnight at 4 °C. The blots were washed, exposed to secondary antibodies and visualized using the ECL system (Perkin, Waltham, MA, US). The quantifications of Rb and p-Rb bands’ intensities were normalized to the corresponding β-actin bands’ intensities. This densitometry analysis was done using HP Deskjet F4180 Scanner with ImageJ software.

### Cell proliferation and soft-agar colony formation assays

LNM35 cells (2 × 10^5^ /well) were plated in 60-mm dishes, and after 24 h transfected with the Akt1-siRNA, Akt2-siRNA or the control sequence (Control-siRNA). At the times indicated, cells were trypsinized and counted using a cell counter (Coulter Counter, Northwell, England). LNM35 cells stably transduced with Akt1-shRNAs (Akt1-shRNA1 & Akt1-shRNA2), Akt2-shRNAs (Akt2-shRNA1 & Akt2-shRNA2) or control sequences (Control-shRNA) were plated at the density of 50,000 cells into six-well tissue culture dishes (50 × 10^3^ cells/well). At the times indicated, cells were trypsinized, collected in 1 ml of medium and counted.

For the colony formation assay, 1 ml per well of 2.4% low melting temperature agar (Bio-Rad) dissolved in distilled water was poured into wells of a 6-well cell culture dish and allowed to set at 4 °C for 5 minutes then incubated at 37 °C for 30 min. A second layer (2.9 ml/well) containing 0.3% of low melting agar dissolved in growth media containing stably transfected cells with Akt1-shRNAs, Akt2-shRNAs or with control-shRNA (5 × 10^3^ cells/ml) was placed on top of the first layer and allowed to set at 4 °C for 5 minutes. After 30 minutes to 1 hour in a humidified incubator at 37 °C, growth medium (2 ml) was added on top of the second layer and then left at 37 °C for 3 weeks. Medium was changed twice a week. At the end of the experiment, colonies were stained for 1 hour with 2% Giemsa stain, and incubated with PBS overnight to remove excess stain. The colonies were photographed and scored.

### Tumor growth assay *in vivo*

Six-week-old athymic NMRI mice (nu/nu, Charles River, Suizfeld, Germany) were maintained under specified pathogen-free conditions. LNM35 cells transiently transfected with Akt2-siRNAs (Akt2-siRNA2) or control sequences (Control-siRNA) (1 × 10^6^ cells) and LNM35 cells stably transduced with Akt2-shRNAs (Akt2-shRNA2) or control sequences (Control-shRNA) (1 × 10^6^ cells) were injected subcutaneously into the lateral flank of the mice. Throughout this study, nude mice were housed in filtered-air laminar flow cabinets and manipulated under aseptic conditions. Tumor dimensions were measured with calipers every week. Tumor volumes (V) were calculated during five weeks using the formula: V = a × b^2^ × 0.4, with “a” being the length and “b” the width of the tumor. The animals were sacrificed five weeks after inoculation. Tumors and axillary lymph node metastases were excised, weighed, and photographed.

Procedures involving animals and their care were conducted in conformity with Institutional guidelines that are in compliance with college of Medicine & Health Sciences, UAE University, national and international laws and policies (EEC Council Directive 86/609, OJ L 358, 1, December 12, 1987; and NIH Guide for Care and Use of Laboratory Animals, NIH Publication No. 85-23, 1985). The animal experiments were performed in accordance with the protocol approved by the animal ethics committee and the Institutional Animal Care at the College of Medicine & Health Sciences/UAE University.

### Wound healing motility and Matrigel invasion assays

LNM35 cells stably transduced with Akt1-shRNAs, Akt2-shRNAs or control-shRNA were grown in six-well tissue culture dishes until confluence. A scrape was made through the confluent monolayer with a plastic pipette tip of 1 mm diameter. Afterwards, the dishes were washed twice and incubated at 37 °C in fresh RPMI containing 10% fetal bovine serum. At the bottom side of each dish, two arbitrary places were marked where the width of the wound was measured with an inverted microscope (objective × 4; Olympus 1 × 71, Japan). Motility was expressed as the mean ± SEM of the distance migrated by the cells at 0, 2, 6, and 24 h.

The invasiveness of the lung cancer cells LNM35 stably transduced with Akt1-shRNAs, Akt2-shRNAs or control-shRNA was tested using BD Matrigel Invasion Chamber (8-μm pore size; BD Biosciences, Le Pont de Claix, France), according to manufacturer protocol. Briefly, 1 × 10^5^ cells in 0.5 ml of media were seeded into the upper chambers of the system, the bottom wells in the system were filled with RPMI 1640 supplemented with 10% fetal bovine serum as a chemo-attractant and then incubated at 37 °C for 24 h. Non-penetrating cells were removed from the upper surface of the filter with a cotton swab. Cells that have migrated through the Matrigel were fixed with 4% formaldehyde, stained with DAPI and counted in 25 random fields under a microscope. The assay was carried out in duplicate and repeated three times for quantitative analysis.

### Transduction of HUVEC cells with Akt1- and Akt2-shRNAs

EndoGROTM Human Umbilical Vein Endothelial Cells (HUVECs) (Millipore, Temecula, CA) were maintained in EndoGROTM-MV-VEGF Complete Media Kit (Millipore, Temecula, CA). HUVEC cells were seeded at 70,000 cells/well into 12-well plates and allowed to attach for 24 h. Cells were incubated with SMARTvector 2.0 Lentiviral shRNA particles targeting Akt1, Akt2 or SMARTvector 2.0 Non-Targeting control particles (Dharmacon Thermo Scientific, US). After 72 h, transduced cells were trypsinized, numbered using a cell counter (Coulter Counter, Northwell, England), and tested for the vascular tube formation.

### Vascular tube formation assay

The Matrigel matrix was thawed, gently mixed to homogeneity using cooled pipettes, and diluted v/v with the EndoGROTM-MV-VEGF Complete Media Kit (Millipore, Temecula, CA, USA). Matrigel, 50 μl/well, supplemented with angiogenic peptides and other effectors was used to coat the wells of 96-well plates. The plate was then incubated for one hour at 37 °C to allow the matrix solution to solidify. The HUVEC cells transduced with Akt1-shRNAs (Akt1-shRNA1 and Akt1-shRNA2), Akt2-shRNAs (Akt2-shRNA1 and Akt2-shRNA2) or control sequences (Control-shRNA) were plated at 40,000 cells per well and incubated for 8 h at 37 °C in 0.1 ml of EndoGROTM-MV-VEGF Complete Media Kit (Millipore, Temecula, CA, USA). The cells were photographed using an inverted phase fluorescence microscope. The tubular network growth area was compared in control-shRNA and Akt1- and Akt2-shRNAs HUVEC cells. Tube formation was quantified by counting the length of tube-like structures formed in each well. The effect of Akt1 and Akt2 silencing on the viability of the HUVECs was determined using a CellTiter-Glo Luminescent Cell Viability assay (Promega Corporation, Madison, USA), as previously described^23^.

### Statistical analysis

Results were expressed as means ± SEM of the indicated data. The difference between experimental and control values were assessed by ANOVA followed by Dunnett’s post-hoc multiple comparison test. For the cell proliferation of Akt-1 and -2 transient silenced cells, tumor growth volume and weight, and ganglia weight data, the difference between experimental and control values were assessed by the unpaired Student’s t-test. *P < 0.05, **P < 0.01 and ***P < 0.001 indicate a significant difference.

## Results

### Validation of Akt1 and Akt2 silencing in LNM35 cancer cells

**Figure 1:**
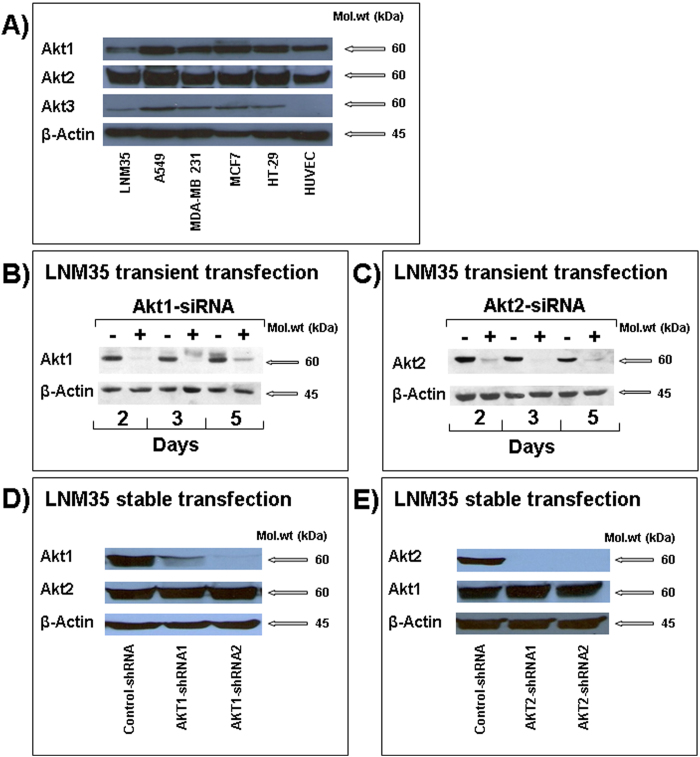
Analysis of selective Akt1 and AKt2 silencing in LNM35 cells. (**A**) Western blot analysis of the three Akt isoform in the human lung cancer cells LNM35 and A549, breast cancer cells MDA-MB-231 and MCF7, colon cancer cells HT29 and the endothelial cells HUVECs. (**B** and **C**) Akt1 and Akt2 protein expression in LNM35 cells transiently transfected with siRNA targeting Akt1 and Akt2 transcript (**+**) respectively or with its siControl oligonucleotide (**−**). Western blot analysis was performed at days 2, 3 and 5 following the transfection. **D and E**) Akt1 and Akt2 protein expression in LNM35 cells stably transduced with control-shRNA and two different designs of Akt1 and AKT2 shRNA (shRNA1 and shRNA2). Data are representative of three independent experiments.

Western blot analysis revealed that the three Akt paralogs are expressed in a panel of cancer and endothelial cells including the lung cancer cells LNM35 and A549, the breast cancer cells MDA-MB-231 and MCF7, the colon cancer cells HT-29 and the endothelial cells HUVEC. We observed a strong expression of Akt1 and Akt2 in these cells whereas the Akt3 signal was weak in all cells and barely detectable in LNM35 and HUVEC cells. In comparison with other cells, LNM35 cells expressed a low level of Akt1 and a very low level of Akt3 (Fig. 1A). We decided to use the highly tumorigenic and metastatic lung cancer cells LNM35 to investigate the impact of specific silencing of the Akt1 and Akt2 isoforms on human lung cancer progression.

To delineate the role of the Akt1 and Akt2 serine/threonine kinases in lung cancer progression, we first used the Akt1 and Akt2 small interfering RNA (siRNA) duplex targeting the human Akt1 and Akt2 transcript respectively^22^. LNM35 cells were transfected with the Akt1-siRNA or Akt2-siRNA (Eurogentec), at 100 nM. Two days after transfections, Akt1 and Akt2 protein levels were efficiently silenced in the LNM35 cells (Fig. 1B,C). Akt1 and Akt-2 silencing remained effective up to 5 days post-transfection. Transfection with the control-siRNA scrambled sequences (−) had no effect on Akt1 and Akt2 protein levels (Fig. 1B,C). Next, LNM35 cells were stably transduced with two designs of SMARTvector 2.0 Lentiviral shRNA particles targeting Akt1 and Akt2. Control cells were transduced with SMARTvector 2.0 Non-Targeting control particles. The puromycin-resistant clones (10 to 12 for each design) were selected and analyzed by western-blot to verify Akt1 and Akt2 silencing. The two different design of shRNA targeting Akt1 and Akt2 (shRNA1, and shRNA2) induced an approximately 90 to 99% decrease in the Akt1 and Akt2 protein level respectively. The selectivity of this silencing was confirmed by the fact that no impact on Akt2 protein was observed in the Akt1 silenced cells, and no impact on Akt1 protein was observed in the Akt2 silenced cells (Fig. 1D,E) and there was no impact on Akt1 and Akt2 protein in the cells transduced with shRNA control particles (control-shRNA) in comparison with parental LNM35 (data not shown).

### Akt2 silencing decreased LNM35 cell proliferation and colony growth in soft agar

**Figure 2:**
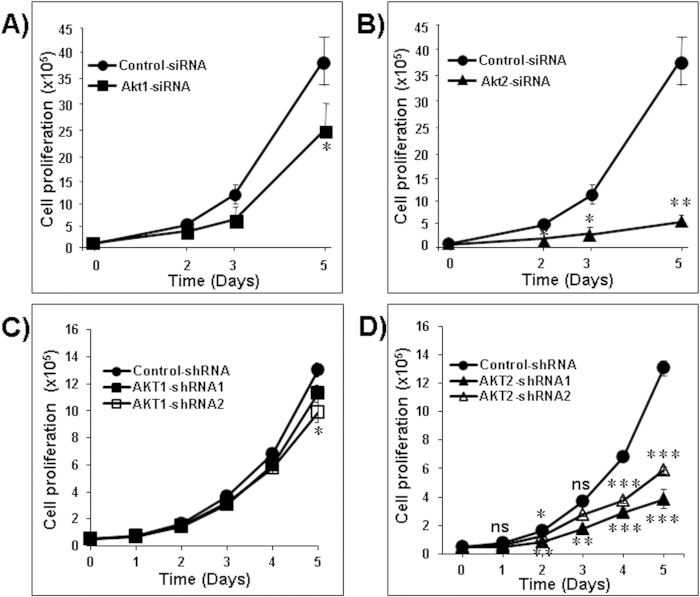
Impact of Akt1 and Akt2 silencing on the proliferation of LNM35 cells. LNM35 cells were transiently transfected with siRNAs targeting the transcripts encoding Akt1, Akt2 or with its siControl sequences. On days 2, 3 and 5 following the transfection, cancer cell proliferation was quantified in silenced Akt1 (**A**) and Akt2 (**B**) cells and compared to the growth curve of the control siRNA cells. LNM35 cells stably transduced by control-shRNA, AKT1-shRNA (Akt1-shRNA1 and Akt1-shRNA2) (**C**) or control-shRNA, AKT2-shRNA (Akt2-shRNA1 and Akt2-shRNA2) (**D**) were seeded into six-well tissue culture dishes (50,000 cells /dish) and counted daily for 5 days. Data are means+/−SEM from 3 independent experiments. Statistical differences obtained at *P < 0.05, **P < 0.01 and ***P < 0.001.

Transient silencing of Akt1 had only a limited impact on cell proliferation. However, transient silencing of Akt2 significantly reduced cell proliferation rates in the LNM35 cells (Fig. 2A,B; *P *<* *0.05*, **P *<* *0.01*), as compared to the corresponding control-siRNA. To confirm the ability of Akt2 to interfere with cancer cell proliferation, control-shRNA cells and their stably silenced counterparts cells Akt1 (shRNA1 and shRNA2) and Akt2 (shRNA1 and shRNA2) were compared for their growth rates. As shown in Fig. 2C,D, stable silencing of Akt1 has little consequence on the proliferation rate of the LNM35 cells. However, Akt2 stably silenced cells exhibit very significantly slower proliferation rates. At day 5, the inhibition was 55 and 70% respectively for Akt2-shRNA1 and Akt2-shRNA2 cells.

**Figure 3:**
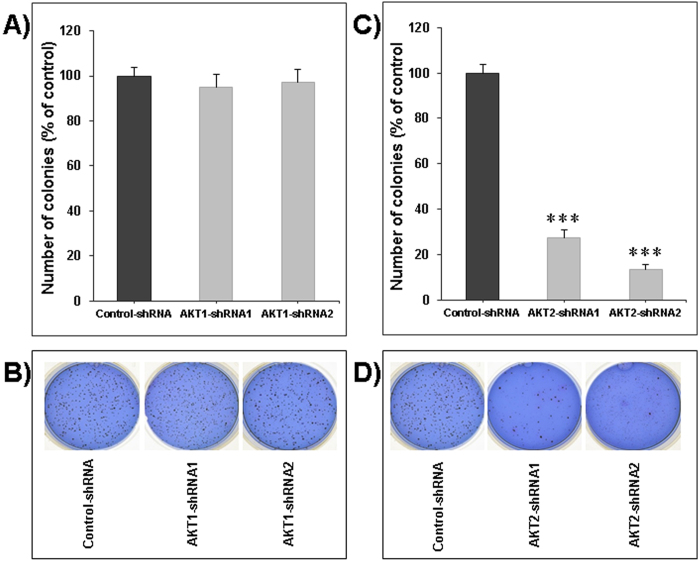
Impact of Akt1 and Akt2 silencing on LNM35 colonies’ growth. Anchorage-independent colonies growth of LNM35 cells stably silenced for Akt1 (**A**) and Akt2 (**C**) is shown. Cells (5×10^3^) were plated in 0.3% soft agar. Three weeks later, colonies were stained with Giemsa and scored. Data are from three experiments performed in duplicate. The colonies formed in soft agar from the two respective silencing designs were photographed after three weeks (**B** and **D**). Data are means +/−SEM from 3 independent experiments. Statistical differences obtained at ***P < 0.001.

Similarly, Akt1 knock-down had no impact on colony formation in soft agar (Fig. 3A,B) whereas Akt2 silencing strongly inhibited the ability of LNM35 cells to form colonies in soft-agar (Fig. 3C,D). The very low impact of Akt1 silencing on cell proliferation and colony growth *in vitro* suggests that it is unlikely that Akt1 has an important function in the growth of LNM35 derived tumors. Our data prompted us to investigate the impact of Akt2 silencing on the growth of LNM35 human lung tumor xenografts in immunodeficient mice.

### Impact of transient and stable Akt2 silencing on tumor growth *in vivo*

**Figure 4:**
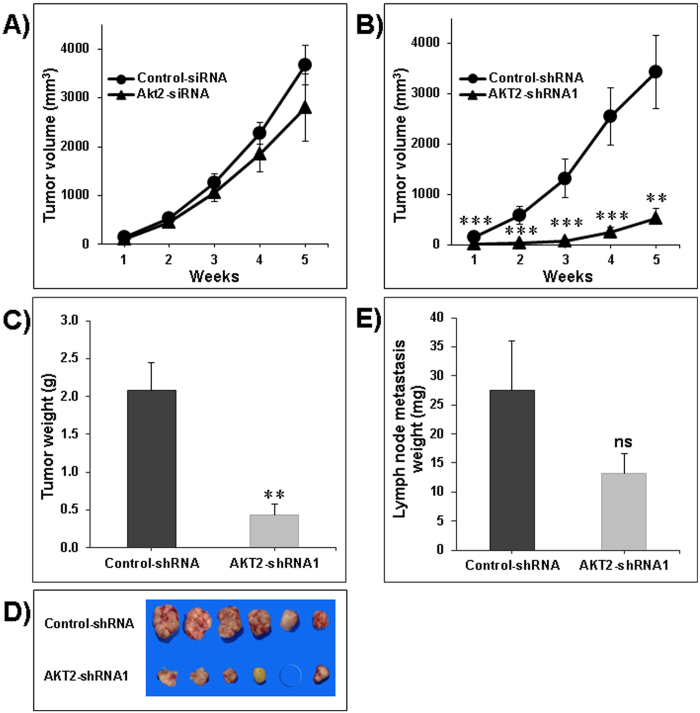
Impact of Akt2 silencing on xenografted tumor growth in nude mice. Nude mice were xenografted subcutaneously into the lateral flank with: (**A**) LNM35 cells transiently transfected twenty four hours before with the siRNA targeting Akt2 (Akt2-siRNA), versus the negative control sequence (Control-siRNA) and (**B**) LNM35 cells stably transduced with Akt2-shRNAs (Akt2-shRNA2) or control sequences (Control-shRNA). Tumor volumes (mm^3^) were measured with caliper every week for a total of five weeks. Mice were then sacrificed and tumors weighed (**C**) and photographed. (**D**) Lymph node metastases of the established LNM35 xenografts were weighed (**E**). Data points represent the mean ± S.E.M of six mice per group. Statistical differences obtained at **P < 0.01, and ***P < 0.001.

After transient transfection with the appropriate siRNA, cancer cells were injected subcutaneously in nude mice. Selective silencing of Akt2 reduced LNM35 tumor volume by 23.7% (2810.1 mm^3^ +/−687.4, p = 0.29), compared with LNM35 cells transfected with control-siRNA (3683 mm^3^ +/−405.6) as measured five weeks after tumor cell injection (Fig. 4A). This mild inhibition of tumor growth may be due to the dilution of siRNA after several cell division cycles. Based on the *in vitro* data showing that both Akt2-shRNA1 and Akt2-shRNA2 significantly inhibited cells’ proliferation and colony growth, we decided to use only one cell line *in vivo* in order to verify the impact of Akt2 silencing on tumor growth. Unlike with the transient silencing, stable silencing of Akt2 strongly inhibited LNM35 tumor growth (532 mm^3^ +/−188, **P *<* *0.01*) in comparison with control-shRNA (3431 mm^3^ +/−725) (Fig. 4B). A similar difference was found in tumor weight at the end of the experiment (0.43 +/−0.14 g versus 2.08 +/−0.37 g, **P *<* *0.01*). No tumor developed in one mouse of the group xenografted with Akt2 silenced cells (Fig. 4C,D). To the best of our knowledge, the LNM35 cell line established by Kozaki *et al*. in 2000 is unique with regard to its 100% incidence of spontaneous lymph node metastasis when injected subcutaneously to nude mice^21^. Using this cellular model, we demonstrated that stable silencing of Akt2 had no impact on the incidence of LNM35 lymph node metastasis but led to the inhibition of their growth (13.3 mg +/−3.37 in the Akt2-shRNA group in comparison with 27.4 mg +/−8.54 in the control-shRNA group). However, this inhibition was not statistically significant (Fig. 4E).

### Effect of Akt2 silencing on the expression and phosphorylation of the retinoblastoma protein (Rb)

**Figure 5:**
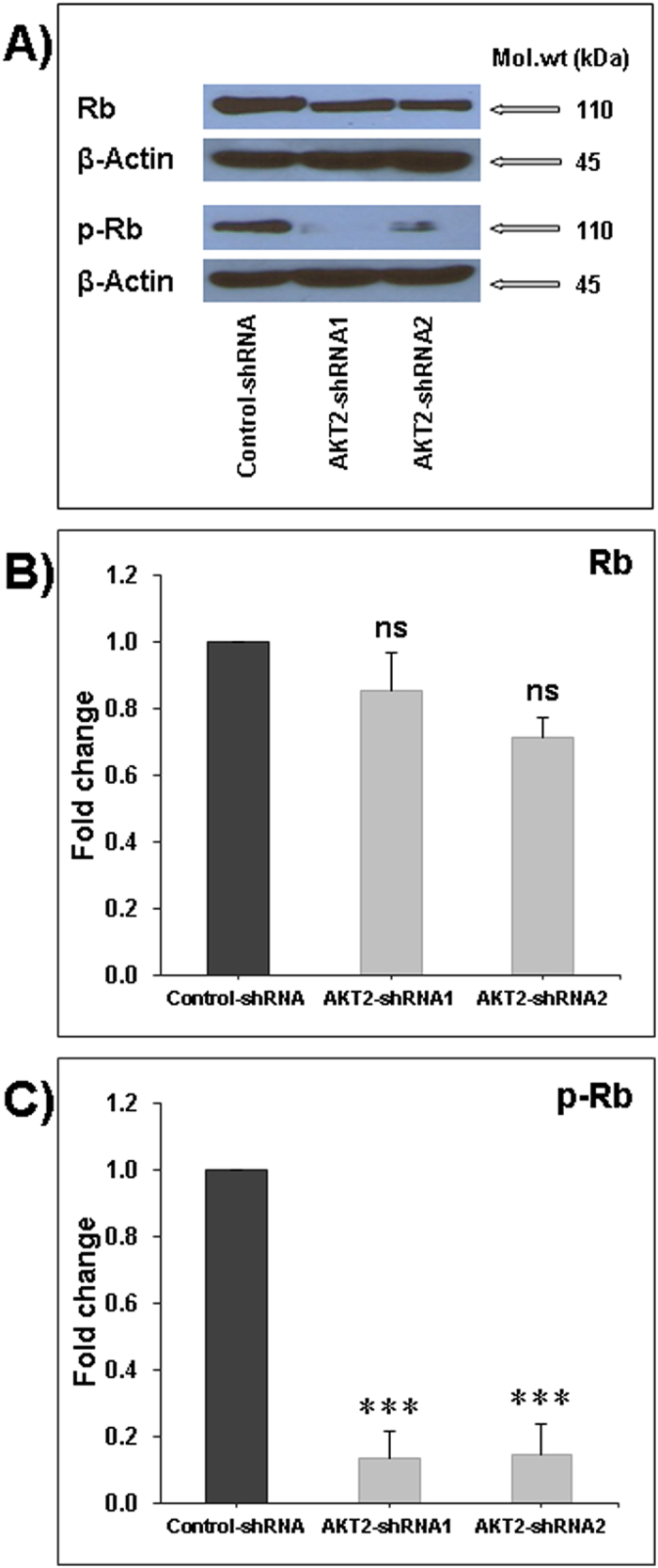
Impact of Akt2 silencing on Rb expression and phosphorylation. (**A**) Representative Western blots of the expression and phosphorylation of Rb in Akt2-shRNA1 and Akt2-shRNA2 cells in comparison with control-shRNA cells. The normalized Rb (**B**) and p-Rb (**C**) bands’ intensities were expressed as fold change in comparison to control samples considered equal to 1. Data are means +/−SEM from 3 independent experiments. Statistical differences obtained with ***P < 0.001.

RB phosphorylation is a key step in the G1/S phase transition. In this context, we demonstrated that the inhibition of cell proliferation, colony formation, and tumor growth resulting from the silencing of Akt2 was correlated with a significant decrease of Rb phosphorylation whereas the total Rb expression was not significantly affected by the knock-down of Akt2 (Fig. 5).

### Akt2 silencing decreases lung cancer cell migration and invasion

**Figure 6:**
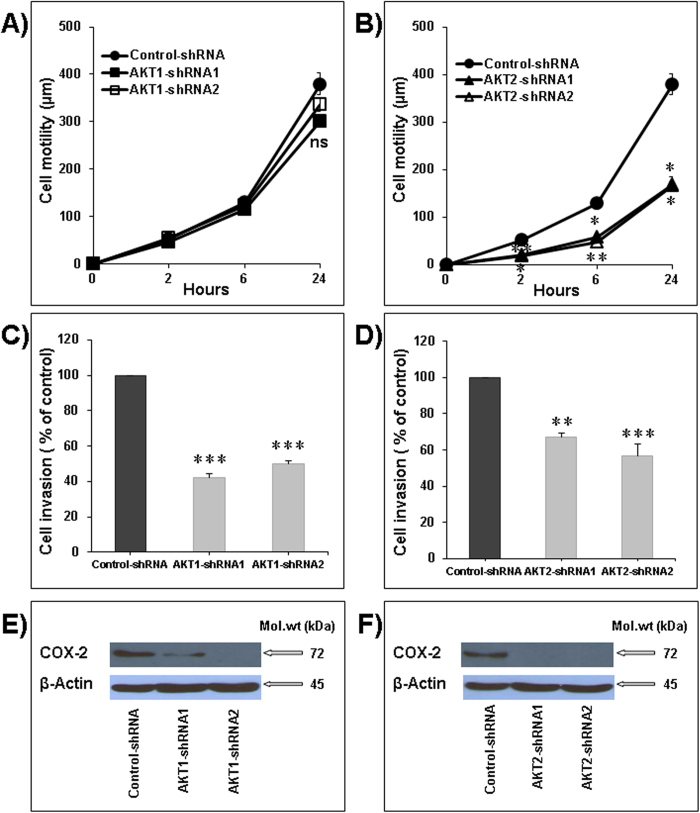
Akt2 silencing impairs cell motility and invasion. (**A** and **B**) Wounds were introduced in respectively Akt1 and Akt2 stably silenced LNM35 confluent monolayers. The mean distance that cells travelled from the edge of the scraped area in 2, 6, and 24 h was measured using an inverted microscope. (**C**) LNM35-Control-shRNA, LNM35-Akt1-shRNA1 and LNM35-Akt1-shRNA2 cells were incubated for 24 h in 0.1% serum. (**D**) LNM35-Control-shRNA, LNM35-Akt2-shRNA1 and LNM35-Akt2-shRNA2 cells were incubated for 24 h in 0.1% serum. Cells that invaded into Matrigel were scored as described in Materials and Methods. (**E**) Western blot analysis of the expression levels of COX-2 in Akt1-shRNA1 and Akt1-shRNA2 cells in comparison with control-shRNA cells. (**F**) Western blot analysis of the expression levels of COX-2 in Akt2-shRNA1 and Akt2-shRNA2 cells in comparison with control-shRNA cells. Data are mean ± S.E.M. from three separate experiments. Statistical differences obtained at *P < 0.05, **P < 0.01 and ***P < 0.001.

First, we examined the effect of Akt1 and Akt2 silencing on cellular motility of the LNM35 cells. Using wound-healing experiments performed with sub-confluent Akt1 (shRNA1 and shRNA2) cells and Akt2 (shRNA1 and shRNA2) cells, we showed that only Akt2 silencing significantly inhibited the cell motility at 2, 6, and 24 h (Fig. 6A,B). Next, we examined the impact of Akt1 and Akt2 silencing on the spontaneous invasiveness of the LNM35 cells. As shown in Fig. 6C,D, stable silencing of Akt 1 and Akt2 significantly decreased cellular invasiveness of the LNM35 cells in Matrigel invasion assay. In addition, we demonstrated that Akt 1 and Akt2 silencing was associated with strongly decreased COX2 expression (Fig. 6E,F). This finding is in agreement with the fact that COX2 plays a significant role in cancer cell migration and invasion^24^. Altogether, these results strongly suggest that both Akt1 and Akt2 plays a role in lung cancer cell invasion and perhaps - by extrapolation - in metastasis.

### Akt2 silencing impairs the formation of capillary-like structures *in vitro*

**Figure 7:**
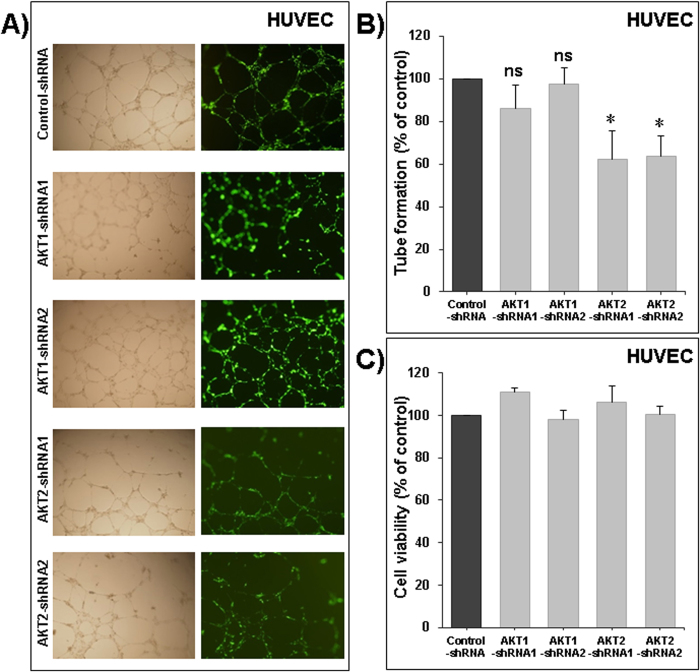
Role of Akt1 and −2 in angiogenesis. (**A**) Formation of capillary-like structures by human umbilical vein endothelial cells (HUVECs) transduced with control-shRNA, two different design of AKT1 shRNA (Akt1-shRNA1 and Akt1-shRNA2) and two different design of AKT2 shRNA (Akt2-shRNA1 and Akt2-shRNA2) and cultured on Matrigel matrix in 96-well plates. (**B**) Quantification of tubular morphogenesis induced in HUVEC cells. Tube formation was determined by the length of tube-like structures containing connected cells. (**C**) Viable cells were assayed as described in Materials and Methods. Data are mean ± S.E.M. from three separate experiments. The “ns” indicate no statistical differences. Statistical differences obtained at *P < 0.05.

Angiogenesis is an attractive target in cancer therapy, because not only is it required for the delivery of oxygen and nutrients for the tumor cells, but also provides a route for metastatic spread of the tumor cells. We showed in Fig. 1A, that Akt 1 and Akt2 are clearly expressed in HUVEC cells. However, Akt3 was almost undetectable. To assess whether Akt1 and -2 plays a role in angiogenesis, we conducted a study on the formation of capillary-like structures *in vitro*, using HUVECs transduced with control-shRNA, Akt1 silenced counterpart’s transfected with Akt1-shRNA1 or Akt1-shRNA2, Akt2 silenced counterpart’s transfected with Akt2-shRNA1 or Akt2-shRNA2. The SMARTvector 2.0 used includes a turboGFP reporter gene to facilitate assessment and optimization of transduction efficiencies. All of our transduced HUVECs were GFP positive (Fig. 7A, right panels). The parental human endothelial cells have the ability to form capillary structures when seeded and cultured on top of Matrigel substrate. Like parental endothelial cells, control-shRNA cells also move from their initial uniform pattern of dispersed cell layers and associate to form a network of cell clusters connected by long, multicellular processes leading to the formation of tube-like structures. Transient silencing of Akt1 had no impact on angiogenesis (Fig. 7A,B) or on HUVECs cell viability (Fig. 7C). However, silencing of Akt2 using Akt2-shRNA1 or Akt2-shRNA2 resulted in a marked inhibition of this spontaneous angiogenic phenotype (Fig. 7A,B). The inhibition of the angiogenic phenotype occurred without reduction of cell viability in Akt2 silenced cells (Fig. 7C). Taken together, these data confirm a strong role of Akt2 in angiogenesis.

## Discussion

The serine/threonine kinase Akt isoforms are over-expressed and activated in many cancers including lung, breast, and colon, but isoform-specific roles in cancer progression still remain unclear. The importance of Akt in tumor growth and progression is indisputable. However, the contradictory results regarding the role of different Akt-isoforms in cancer progression published by different research groups do not answer the question which isoform is the most promising target for cancer therapy. In this study, we demonstrate that the Akt2 isoform plays an important role in lung cancer cell proliferation, colony, and tumor growth, as well as in motility, invasion, and angiogenesis. In our cellular model, we also demonstrated that Akt1 isoform is mainly involved in cellular invasion.

Our data demonstrate that Akt2 is necessary for lung cancer cell proliferation, colony and tumor growth in accordance with the decrease in Rb phosphorylation. Numerous reports from other laboratories are in agreement with our conclusions. It has been reported that suppression of Akt2 expression in human lung adenocarcinoma cell line A549 resulted in notable inhibition of cell proliferation and colony growth^25^. Similarly, silencing of Akt2 in neuroblastoma and gliomas impaired cell proliferation and colony growth^26^,^27^. It has also been reported that loss of Akt2 decreased the proliferation of Pten wild-type astrocytes^28^. In breast cancer, it has been demonstrated that Akt2 is the most relevant isoform to cell proliferation and colony growth^29^ and the stable ablation of Akt2 in mammary tumors of MTB-IGFIR transgenic mice delayed tumor onset and growth rate^30^. It has also been reported that silencing of Akt2 resulted in decreased IGF-IR mediated cell proliferation^31^ as well as ovarian and hepatocellular carcinoma cell proliferation and growth^32^. In another study, transient silencing of Akt2 had a small but non-significant impact on H460 colony growth^33^. It has also been reported that in fibroblasts Akt2 has no function in cell proliferation^34^. On the contrary, a very recent study using a viral oncogene-induced mouse model of lung cancer, showed a dramatic increase in tumorigenesis in Akt2-/- mice due to the enhancement of cell proliferation and inhibition of apoptosis^35^. Our results are in agreement with the large majority of studies suggesting that Akt2 plays a major role in cancer cell proliferation and consequently in tumor growth. However, it remains possible that, depend on cancer cell types, different Akt isoforms may be involved in cellular proliferation and tumor growth.

Our data are also in line with a very recent publication indicating that the inhibition of cell proliferation by the PI3K/AKT inhibitor LY294002 was, at least in part, mediated by the decrease in Rb phosphorylation^36^. In this context, another study reported the hypo-phosphorylation of Rb in Akt2-depleted cells along with a decrease in Rb expression level^37^.

We also show that Akt1 has only a minimal role in LNM35 lung cancer cell proliferation and has no impact in colony growth. Similarly, it has been reported that silencing of Akt1 resulted in a moderate inhibition of A549 human lung adenocarcinoma cell proliferation and colony growth^25^. However, in a transient Akt1 silencing study, Akt1 had a marked role in A549 and NCI-H460 NSCLC colony growth^33^. It has also been reported that deletion of Akt1, but not Akt2 prevented lung tumor progression in a tobacco carcinogen-induced model and in a genetic mutant K-ras model^38^. This study also reported that depletion of Akt2 induced smaller lung tumor size although the difference was not statistically significant and concludes that the role of Akt2 in lung tumorigenesis cannot be excluded. In fibroblasts, only Akt1 was required for cellular proliferation, while Akt2 played no role^34^. Akt-1 deficiency was also shown to reduce murine mammary epithelial tumor cell proliferation rate^39^. Conversely, another report indicated that neither Akt1 nor Akt2 are required for HeLa cell proliferation on extracellular matrix^22^. In agreement with our study, it has been reported that only Akt2 silencing was able to reduce ovarian cell proliferation whereas Akt1 silencing did not affect cell growth^32^.

Lung cancer patients are at high risk of recurrence in the form of metastatic disease. Metastasis starts with the acquisition of a scattered phenotype and cell migration within the primary tumor, leading to local tissue invasion and entry into lymph or blood vessels and finally colonization of distant organs. We demonstrate that Akt2 is a key signaling kinase for lung cancer cell motility, invasion, and for angiogenesis *in vitro*. Accordingly, it has been reported that suppression of Akt2 expression in human lung adenocarcinoma cell line A549 resulted in a notable inhibition of cellular invasion^25^. Akt2 was also demonstrated to be strongly involved in the migration of NSCLC-derived disseminated tumor cells^40^. It has also been found that Akt2 enhances the motility and the invasiveness of breast, ovarian, and pancreatic cancer^31^,^41^,^42^. Another study indicates that cellular invasion of skin esophageal fibroblasts was attenuated by silencing of Akt2^43^. Similarly, silencing of Akt2 in neuroblastoma impaired cellular migration and invasion and decreased liver metastasis^26^. Akt2 also plays a significant role in colon cancer metastasis^44^. On the contrary, it has been reported that down-regulation of Akt2 in human prostate cancer cells PC3 enhanced cellular migration and invasion^45^. Furthermore, another study on NSCLC suggested that silencing of Akt2 has no significant impact on cell migration^33^. Discordant data have been reported about the role of Akt2 in cancer cell migration and invasion, but majority are in agreement with our current data indicating that Akt2 is required for the invasive potential of lung cancer cells.

Our results also show that silencing Akt-1 had no significant impact on LNM35 cell migration but significantly decrease cellular invasion. Cell migration is a complex process and although Cox2 is involved in its regulation, there may be other (Akt2-dependent) pathways involved that compensate for the reduced activity of Cox2 in the case of Akt1 knock-down. Irie and colleagues reported that silencing Akt1 enhanced non-transformed breast epithelial cells’ migration induced by IGF-1 or EGF, in contradiction with another work showing that Akt1 increased mammary tumor cells migration across the endothelial cell barrier and that Akt1 deficiency reduced lung metastasis^31^,^39^. The decrease in cell invasion due to the Akt1 knock-down is in agreement with another study reporting that silencing of Akt1 resulted in a moderate inhibition of A549 human lung adenocarcinoma cell invasion^25^. Recently, it has been reported that in fibroblasts Akt1 and Akt2 play opposite roles in the regulation of podosome formation and the extracellular matrix degradation leading to invasion. These authors also reported that the roles of these Akt isoforms are not only cell dependent but also experimental context dependent^46^.

It has been previously reported that COX-2 expression is significantly increased in LNM35 cells suggesting the potential involvement of COX-2 in the invasiveness and metastasis^21^. Also, it has been demonstrated recently that Akt regulates COX-2 protein expression in human endometrial cancer cells^47^. In the current study we demonstrate that the decrease in the invasiveness of Akt1- and Akt2-silenced cells was associated with a significant decrease in COX2 expression.

In conclusion, we believe in the potential pharmacological impact of selective Akt2 inhibitors to decrease lung tumor growth as well as cell invasion and metastasis even true that Akt1 also play a role in cancer cell invasion.

## Additional Information

**How to cite this article**: Attoub, S. *et al*. Akt2 knock-down reveals its contribution to human lung cancer cell proliferation, growth, motility, invasion and endothelial cell tube formation. *Sci. Rep*. **5**, 12759; doi: 10.1038/srep12759 (2015).
