# Author Correction: Akt2 knock-down reveals its contribution to human lung cancer cell proliferation, growth, motility, invasion and endothelial cell tube formation

**DOI:** 10.1038/s41598-022-14136-7

**Published:** 2022-06-15

**Authors:** Samir Attoub, Kholoud Arafat, Nasseredine Kamel Hammadi, Jan Mester, Anne-Marie Gaben

**Affiliations:** 1 Department of Pharmacology and Therapeutics, College of Medicine & Health Sciences, UAE University, P. O. Box 17666, Al Ain, United Arab Emirates; 2 INSERM U673 and U938, Molecular and Clinical Oncology of Solid Tumors, University Pierre and Marie Curie Paris VI, Saint-Antoine Hospital, 75571 Paris Cedex 12, France

Correction to: *Scientific Reports* 10.1038/srep12759, published online 03 August 2015

This Article contains an incorrect image in Figure 7A. As a result of a mistake during figure assembly, in Figure 7A, left column, an incorrect image for AKT1-shRNA2 was used; the image originates from another sample, AKT1-shRNA1. The correct panel 7A is shown below as Figure 1.

**Figure 1:**
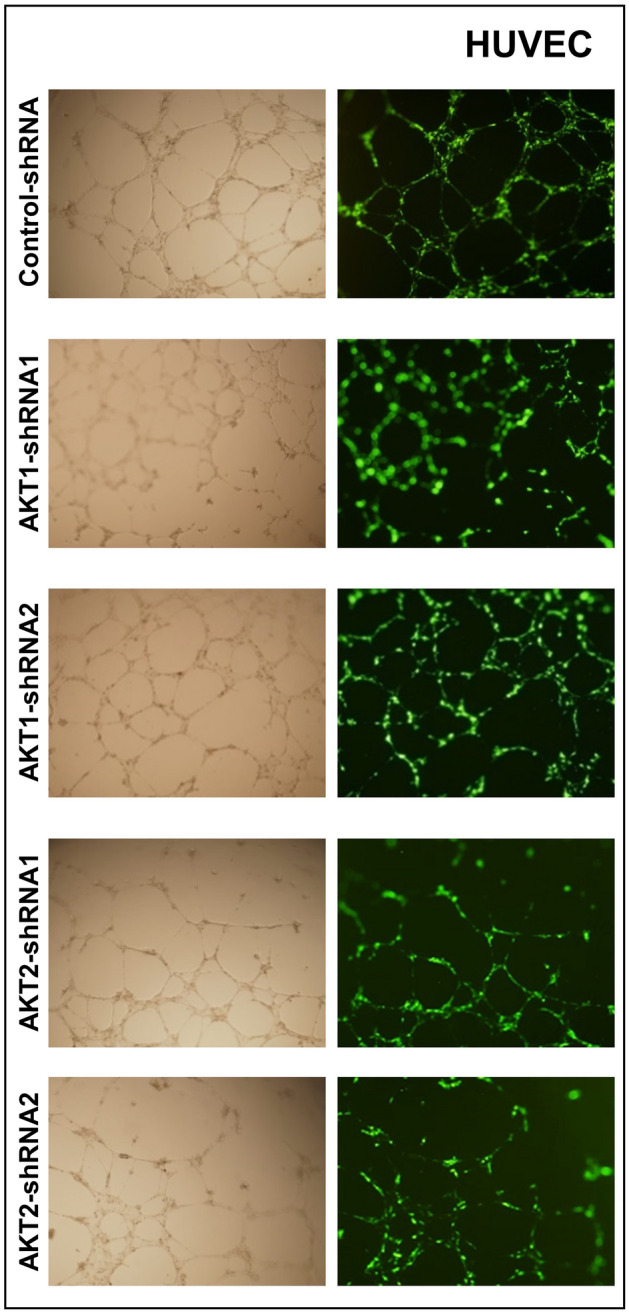
Corrected panel 7A: Formation of capillary-like structures by human umbilical vein endothelial cells (HUVECs) transduced with control-shRNA, two different design of AKT1 shRNA (Akt1-shRNA1 and Akt1-shRNA2) and two different design of AKT2 shRNA (Akt2-shRNA1 and Akt2-shRNA2) and cultured on Matrigel matrix in 96-well plates.

This change does not affect the conclusions of the Article.

